# Total and Added Sugar Intakes Are Increasing among Children and Adolescents in China: Findings from CHNS 1997–2011

**DOI:** 10.3390/nu14163340

**Published:** 2022-08-15

**Authors:** Yan Liu, Jing Cheng, Lijin Wan, Wei Chen

**Affiliations:** 1 Department of Clinical Nutrition, Peking Union Medical College Hospital, Chinese Academy of Medical Sciences, No. 1 Shuai Fu Yuan, Dong Cheng District, Beijing 100730, China; 2 Division of Oral Epidemiology & Dental Public Health, University of California at San Francisco, 3333 California Street, Ste. 495, San Francisco, CA 94143, USA; 3 Baidu Inc., No. 10 Xibeiwang East Road, Beijing 100193, China

**Keywords:** total sugar, added sugar, time trends, dietary sources, children, adolescents

## Abstract

A high level of sugar intake has been linked to poor dietary quality and a wide range of chronic diseases. However, data on sugar intake are still scarce in China. This study aimed to provide time trends in the total and added sugar intakes among Chinese children and adolescents from 1997 to 2011. A nationwide ongoing open prospective cohort study of Chinese children and adolescents aged 3–17 years (*n* = 13,212) was conducted by using data from the China Health and Nutrition Survey (CHNS) 1997–2011 (six 1-year cycles). An individual dietary intake was collected from three consecutive 24-h recalls during randomly allocated home visits. Data for total and added sugar contents were determined based on the U.S. Department of Agriculture (USDA) National Nutrient Database for Standard Reference, Release 28 (SR28), the Food Patterns Equivalents Database (FPED) 2015–2016, and the labeled ingredients and nutrient contents. General linear regression was used to estimate time trends. Over the 15-year period, total sugar and added sugar intakes increased among all age groups studied (3–17 years: total sugar increased from 11.2 ± 0.3 g/d to 28.1 ± 0.5 g/d, added sugar increased from 1.0 ± 0.1 g/d to 7.2 ± 0.3 g/d; 3–6 years: 9.5 ± 0.6 g/d to 25.1 ± 0.9 g/d, 1.3 ± 0.2 g/d to 6.9 ± 0.4 g/d; 7–12 years: 11.4 ± 0.5 g/d to 28.1 ± 0.8 g/d, 0.9 ± 0.1 g/d to 7.1 ± 0.5 g/d; 13–17 years: 11.8 ± 0.4 g/d to 31.4 ± 1.1 g/d, 1.0 ± 0.2 g/d to 7.6 ± 0.6 g/d) (all *p* for trend < 0.001). Adolescents aged 13–17 years had the highest total sugar intake, and children aged 3–6 years had the highest added sugar intake, except for 2011. Children and adolescents living in urban areas and who were overweight had much higher total and added sugar intakes than those residing in rural areas and of non-overweight/obesity. Furthermore, the dietary sources of total and added sugars have become more diverse over the study period. In conclusion, we observed a notable rise in total and added sugar intakes among children and adolescents across all age groups, both genders, both urban and rural areas, and all BMI categories, with dietary sources of total and added sugars becoming more diverse in China over 15 years.

## 1. Introduction

A high level of sugars intake has been linked to poor dietary quality [1] and a wide range of chronic diseases, such as dental caries [2], obesity [3], type 2 diabetes [4], hepatic steatosis [5,6], cardiovascular diseases [7], and metabolic syndrome [4]. However, although both the World Health Organization (WHO) and the Dietary Guidelines for Americans (DGA) 2020–2025 recommended that free sugars or added sugars intake should be limited to less than 10% of total energy intake per day [1,8], excessive sugar intakes remain a global public health issue. In the United States, residents ≥ 2 years obtained 14.6% of their total energy intake from added sugars in 2007–2008 [9], which was still a pressing concern even in 2016 [8]. A similar problem has also been observed among German 3–18-year-olds throughout a 30-year study period [10].

In addition to a higher sweet preference than adults [11], children and adolescents are at a “critical period” for the development of various diseases in later life [12,13]. Therefore, they are particularly vulnerable to excessive sugar intake and the corresponding long-term health risks. On the contrary, establishing a healthy dietary pattern early in life may have a significant beneficial impact on food choices, health promotion, and disease prevention over the course of decades [8]. Thus, more attention is needed to investigate and then regulate sugar intake among children and adolescents. However, data on the time trend of the total and added sugar intakes are still scarce in China, preventing targeted health education and policymaking for improving sugar intake in these vulnerable groups.

This study aimed to estimate total and added sugar intakes and time trends among Chinese children and adolescents aged 3–17 years, covering 15 years from 1997 to 2011, and using data from three consecutive 24-h recalls collected by the China Health and Nutrition Survey (CHNS).

## 2. Methods

### 2.1. Study Sample

Data from the CHNS was used to examine the trend of sugar intake over chosen years in China. The CHNS is a nationwide ongoing open prospective cohort that was initiated in 1989, including a sample of about 7200 households with over 30,000 individuals from 15 provinces and municipal cities of China [14]. The CHNS rounds were conducted every 2–4 years. The complex sampling methodology used in CHNS has been described elsewhere [15]. Children and adolescents aged 3–17 years, who participated in CHNS between 1997 and 2011 with dietary information for two or more days (*n* = 13,212), were included in the analyses of this study. Since the CHNS is an international collaborative project between the National Institute for Nutrition and Health (NINH) at the Chinese Center for Disease Control and Prevention (CCDC) and the Carolina Population Center at the University of North Carolina at Chapel Hill (UNC), it was approved by the ethical review committees of the CCDC and the UNC. Signed, informed consent was obtained from all participants or their proxy respondents before the surveys.

### 2.2. Assessment of Sugar Intakes and Dietary Sources

Individual dietary intake was collected from three consecutive 24-h recalls during randomly allocated home visits from Monday to Sunday. Trained and experienced interviewers coached the participants (≥12 years old) or their proxy respondents (<12 years old) to carefully report all food consumed both at home and away from home by using a standardized protocol. Household food inventory and in-house weighing approach were used to check the quality of the 24-h recall data. The nutrient content of foods consumed each day, including intakes of total calories, carbohydrates, fats, and proteins, was determined by CHNS using the 1991 China Food Composition Tables (FCTs) [16] in surveys before 2004 and updated version of FCTs (2002/2004) [17,18] in the following waves (2004–2011).

Total and added sugar intakes were determined based on the U.S. Department of Agriculture (USDA) National Nutrient Database for Standard Reference, Release 28 (SR28) [19] and the Food Patterns Equivalents Database (FPED) 2015–2016 [20]. Specifically, items with exact food-description matches were assigned the value of total and added sugars. Independent estimates for food items without an exact match were imputed by 2 investigators (Y.L. and L.W.). Processed foods that had no similar comparison food in the SR28 or FPED 2015–2016 data set but were sold in supermarkets in San Francisco were estimated by the labeled ingredients and nutrient contents. To obtain the amount of total and added sugars consumed in each food, we converted the teaspoon equivalents to grams by multiplying by 4.2 g/teaspoon [20] and then multiplied the total amount (in grams) of each food by the content of the total or added sugars in the corresponding food (grams/100 g). Subsequently, we calculated the average daily intake of them for each participant. These results were multiplied by 4 kcal and then divided by the total energy intake (in kcal/d) to obtain the percentage of total energy (%E) from total or added sugars. We adopted the DGA’s cut-off value to categorize the added sugar (%E) of children and adolescents [8]. Each food or beverage was categorized based on the food or beverage groups of the FCTs (1991/2002/2004) [16,17,18], and the dietary sources of total and added sugars (percentage contribution of each food or beverage group to total and added sugar intakes) were then calculated.

### 2.3. Demographic Variables

Self-reported demographic information, such as date of birth, gender, annual household income, and medical insurance status was obtained by a structured interview. Area of residence, categorized as urban (cities and towns) and rural (outlying areas), was also collected. 

### 2.4. Anthropometric Measurements

The weight and height of participants were measured based on the WHO standard using calibrated beam scales and portable stadiometers, respectively. Body mass index (BMI) was defined as weight/height^2^ (kg/m^2^). Overweight and obesity were determined according to the age- and gender-specific BMI cut-off values proposed by the International Obesity Task Force (IOTF) [21].

### 2.5. Statistical Analysis

To compare the basic demographics and characteristics of children and adolescents with different levels of total sugar intake, the participants were divided into Q1 and Q2 groups by the median value of total sugar intake of the corresponding characteristic subgroup. Continuous variables (age, BMI, dietary intakes, and annual household income, etc.) were summarized with means and standard errors (SEs), and categorical variables (age groups, gender, residential area, and medical insurance, etc.) with frequency and percentage. Student’s *t*-test, Mann–Whitney U test, and chi-squared test were used to examine differences in characteristics between groups. The trends of total and added sugar intakes, as well as total sugar (%E) and added sugar (%E) over years, both overall and by the characteristic groups, were graphed and fitted, respectively, with a general linear regression model. Sampling weights were incorporated and correlation within strata were accounted for in all the analyses. All *p* values were two-sided, and statistical significance was determined at *p* < 0.05 (two-tailed tests). All analyses were conducted with International Business Machines (IBM) Statistical Package for the Social Sciences (SPSS) version 26.0 (IBM Corp., Armonk, NY, USA).

## 3. Results

### 3.1. Participant Basic Demographics and Characteristics by Years

The basic demographics and characteristics of Chinese children and adolescents by years from 1997–2011 are provided in Table 1. The average age increased from 1997 to 2000, peaked, and then continued to decline. The BMI ranged from 17.0 ± 0.1 kg/m^2^ in 1997 to 17.9 ± 0.1 kg/m^2^ in 2011. Over the study period, the total energy intake decreased from 1844.3 ± 11.0 kcal/d to 1471.4 ± 12.6 kcal/d. Although total fat intake fluctuated, fat intake (E%) increased year by year. Likewise, total protein intake decreased, whereas total protein (%E) increased from 12.1 ± 0.0% to 14.0 ± 0.1%. Both the total carbohydrate intake and total carbohydrate (%E) declined, however, total sugar intake, total sugar (%E), added sugar intake, and added sugar (%E) increased from 11.2 ± 0.3 g/d to 28.1 ± 0.5 g/d, 2.6 ± 0.1% to 7.8 ± 0.1%, 1.0 ± 0.1 g/d to 7.2 ± 0.3 g/d, and 0.2 ± 0.0% to 1.9 ± 0.1%, respectively. Consistent with the upward trends in the total and added sugar intakes, the percentages above or equal to the DGA’s cut-off value for the added sugar intake also increased (Supplementary Table S1). Annual household incomes also increased substantially during the time span covered in the present study. In addition, the percentage of boys and girls was stable over the study period, but consistently higher for boys than for girls. The percentage of children and adolescents living in urban areas, who were overweight or obese, and with medical insurance increased.

### 3.2. Comparisons of Basic Demographics and Characteristics by Total Sugar Intake Levels

The comparisons of the basic demographics and characteristics of Chinese children and adolescents with different total sugar intakes are provided in Table 2. The Q2 group showed a significantly higher BMI, total energy intake, total fat intake, total fat (%E), total protein intake, total protein (%E), added sugar intake, added sugar (%E), and annual household income, and a lower total carbohydrate intake and total carbohydrate (%E) than the Q1 group in most age and gender subgroups (*p* < 0.05). Children and adolescents living in urban areas, who were overweight or obese, and with medical insurance had greater proportions in the Q2 group, whereas the opposite is true for those residing in rural areas, of non-overweight/obesity, and without medical insurance in most age and gender subgroups (*p* < 0.05).

### 3.3. Trends in Total and Added Sugars Intake by Age Groups

**Figure 1:**
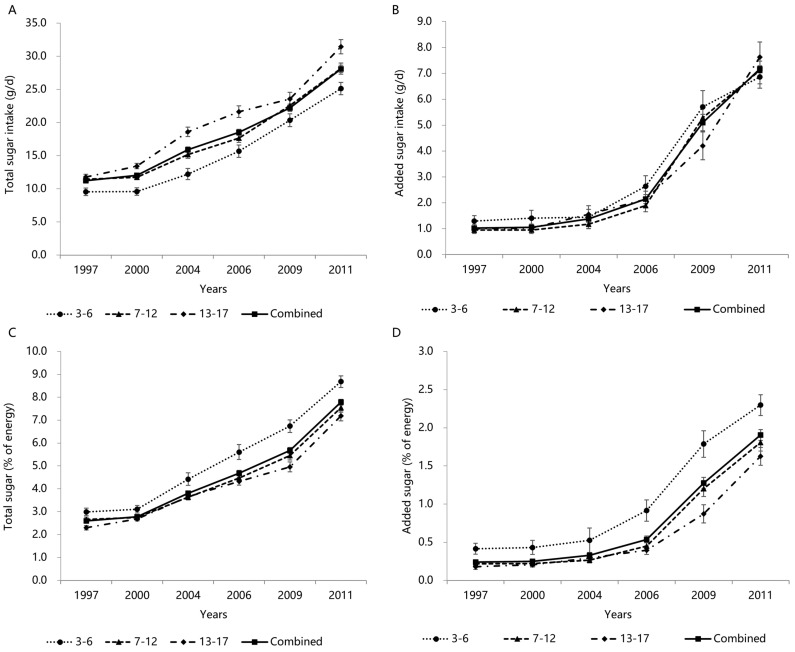
Mean (±SE) trends in total sugar intake (**A**), added sugar intake (**B**), total sugar (%E) (**C**), and added sugar (%E) (**D**) in Chinese children and adolescents by age group, CHNS 1997–2011. All results of linear trends by using general linear regression were *p* < 0.001.

Trends in the total sugar intake, added sugar intake, total sugar (%E), and added sugar (%E) by age groups from 1997–2011 are presented in Figure 1A–D. Over the 15-year period, the total sugar intake (A) and total sugar (%E) (C) increased among all age groups studied [3–6 years (1997 to 2011): 9.5 ± 0.6 g/d to 25.1 ± 0.9 g/d, 3.0 ± 0.2% to 8.7 ± 0.3%; 7–12 years (1997 to 2011): 11.4 ± 0.5 g/d to 28.1 ± 0.8 g/d, 2.7 ± 0.1% to 7.5 ± 0.2%; 13–17 years (1997 to 2011): 11.8 ± 0.4 g/d to 31.4 ± 1.1 g/d, 2.3 ± 0.1% to 7.2 ± 0.2%] (all *p* for trend < 0.001), most notably since 2000. The total sugar intake was higher in adolescents aged 13–17 years than in participants in other age groups, but children aged 3–6 years had the highest total sugar (%E). Similar trends were observed in the added sugar intake (B) and added sugar (%E) (D) [3–6 years (1997 to 2011): 1.3 ± 0.2 g/d to 6.9 ± 0.4 g/d, 0.4 ± 0.1% to 2.3 ± 0.1%; 7–12 years (1997 to 2011): 0.9 ± 0.1 g/d to 7.1 ± 0.5 g/d, 0.2 ± 0.0% to 1.8 ± 0.1%; 13–17 years (1997 to 2011): 1.0 ± 0.2 g/d to 7.6 ± 0.6 g/d, 0.2 ± 0.0% to 1.6 ± 0.1%]. Both the added sugar intake and % of energy increased substantially after 2006 in all age groups. Children aged 3–6 years had the highest added sugar intake than participants in other age groups over the years, except for the added sugar intake in 2011.

### 3.4. Trends in Total and Added Sugar Intake by Gender

**Figure 2:**
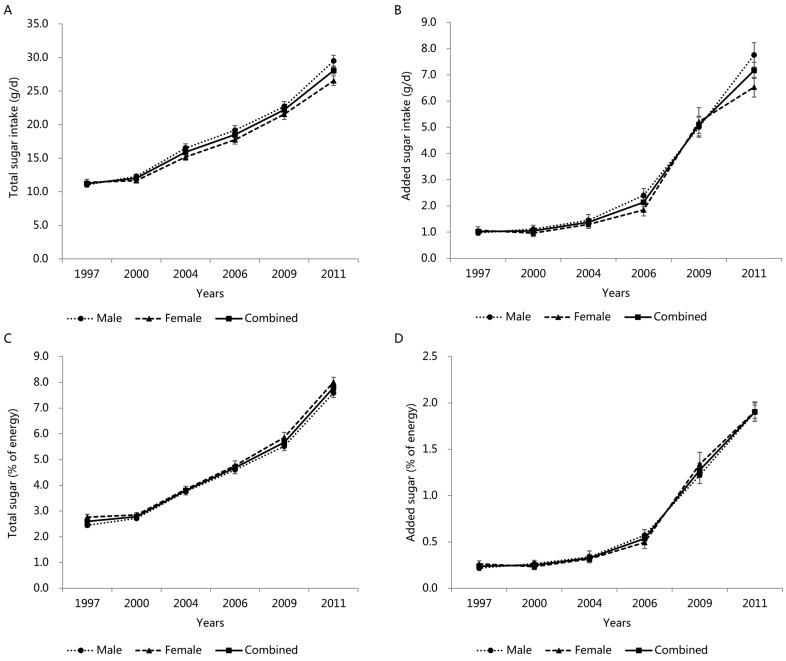
Mean (±SE) trends in total sugar intake (**A**), added sugar intake (**B**), total sugar (%E) (**C**), and added sugar (%E) (**D**) in Chinese children and adolescents by gender, CHNS 1997–2011. All results of linear trends by using general linear regression were *p* < 0.001.

Figure 2A–D shows the trends in the total sugar intake, added sugar intake, total sugar (%E), and added sugar (%E) by gender from 1997–2011. All the variables increased in both genders over the study period (all *p* for trend < 0.001), which were relatively close between boys and girls [boys (1997 to 2011): total sugar intake 11.1 ± 0.4 g/d to 29.5 ± 0.8 g/d, added sugar intake 1.0 ± 0.1 g/d to. 7.8 ± 0.5 g/d, total sugar (%E) 2.5 ± 0.1% to 7.6 ± 0.2%, 0.2 ± 0.0% to 1.9 ± 0.1%; girls (1997 to 2011): 11.4 ± 0.5 g/d to 26.5 ± 0.7 g/d, 1.1 ± 0.1 g/d to 6.5 ± 0.4 g/d, 2.8 ± 0.1% to 8.0 ± 0.2%, 0.3 ± 0.0% to 1.9 ± 0.1%].

### 3.5. Trends in Total and Added Sugar Intake by Area

**Figure 3:**
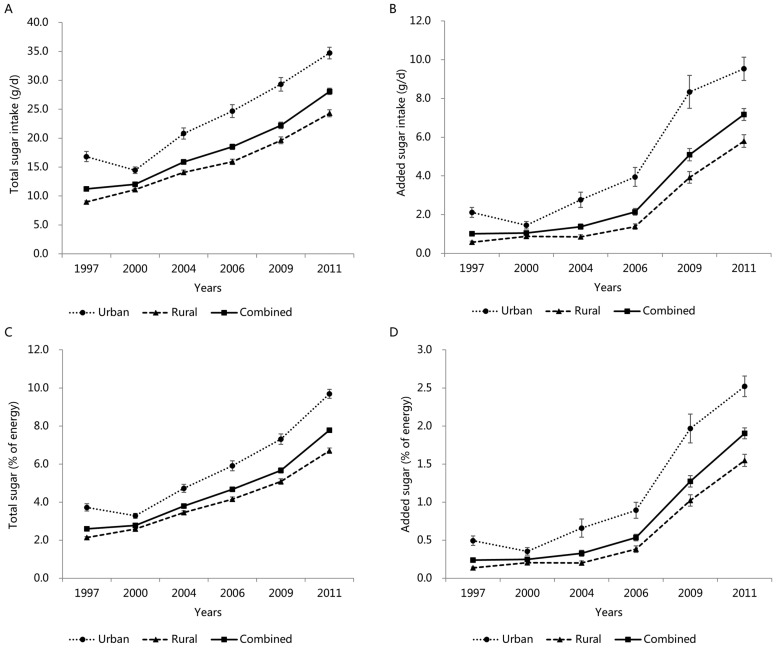
Mean (±SE) trends in total sugar intake (**A**), added sugar intake (**B**), total sugar (%E) (**C**), and added sugar (%E) (**D**) in Chinese children and adolescents by area, CHNS 1997–2011. All results of linear trends by using general linear regression were *p* < 0.001.

The growth trends in the total sugar intake, added sugar intake, total sugar (%E), and added sugar (%E) by area over six cycles (1997–2011) are shown in Figure 3A–D [urban area (1997 to 2011): 16.8 ± 0.9 g/d to 34.7 ± 1.0 g/d, 2.1 ± 0.3 g/d to 9.5 ± 0.6 g/d, 3.7 ± 0.2% to 9.7 ± 0.2%, 0.5 ± 0.1% to 2.5 ± 0.1%; rural area (1997 to 2011): 8.9 ± 0.2 g/d to 24.3 ± 0.6 g/d, 0.6 ± 0.1 g/d to 5.8 ± 0.3 g/d, 2.1 ± 0.0% to 6.7 ± 0.1%, 0.1 ± 0.0% to 1.5 ± 0.1%] (all *p* for trend < 0.001). These four variables for children and adolescents living in urban areas were higher than those residing in rural areas.

### 3.6. Trends in Total and Added Sugar Intake by BMI Categories

**Figure 4:**
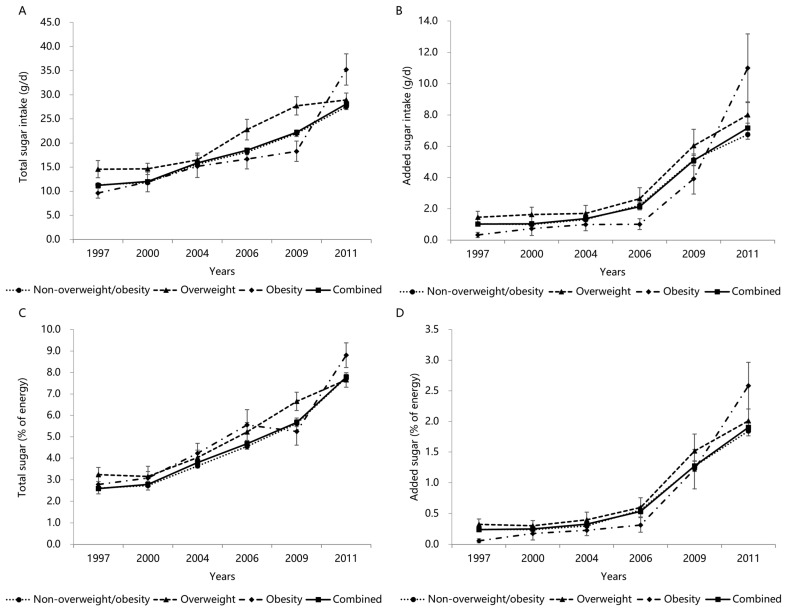
Mean (±SE) trends in total sugar intake (**A**), added sugar intake (**B**), total sugar (%E) (**C**), and added sugar (%E) (**D**) in Chinese children and adolescents by BMI categories, CHNS 1997–2011. All results of linear trends by BMI categories using general linear regression were *p* < 0.001.

The upward trends in the total sugar intake, added sugar intake, total sugar (%E), and added sugar (%E) by the BMI categories over 15 years are shown in Figure 4A–D [non-overweight/obesity (1997 to 2011): 11.3 ± 0.3 g/d to 27.5 ± 0.6 g/d, 1.0 ± 0.1 g/d to 6.7 ± 0.3 g/d, 2.6 ± 0.1% to 7.8 ± 0.1%, 0.2 ± 0.0% to 1.8 ± 0.1%; overweight (1997 to 2011): 14.6 ± 1.8 g/d to 28.9 ± 1.5 g/d, 1.5 ± 0.4 g/d to 8.0 ± 0.8 g/d, 3.2 ± 0.3% to 7.7 ± 0.3%, 0.3 ± 0.1% to 2.0 ± 0.2%; obesity (1997 to 2011): 9.6 ± 1.1 g/d to 35.2 ± 3.2 g/d, 0.3 ± 0.2 g/d to 11.0 ± 2.2 g/d, 2.8 ± 0.4% to 8.8 ± 0.6%, 0.1 ± 0.0% to 2.6 ± 0.4%] (all *p* for trend < 0.001). Children and adolescents who were overweight had a higher total sugar, added sugar, total sugar (%E), and added sugar (%E) intake than those of non-overweight/obesity. Interestingly, the total sugar, added sugar, and added sugar (%E) were lower in obese children and adolescents than those in other BMI categories between 1997 and 2009, while the opposite was true in 2011.

### 3.7. Trends in Total and Added Sugar Intake by Gender, Area, and BMI Categories among Different Age Groups

The trends in the total and added sugar intakes by gender, area, and BMI categories in different age groups (3–6, 7–12, 13–17) from 1997 to 2011 are shown in Table 3. In all age groups, these variables in all subgroups showed the same upward trends as the results in the age-combined analysis (all *p* for trend < 0.001).

### 3.8. Dietary Sources of Total and Added Sugars

Table 4 shows the dietary sources of the total and added sugars among Chinese children and adolescents in 1997 and 2011. Consistent with the upward trends in the total and added sugars intake, the dietary sources of the total and added sugars have become more diverse over the last 15 years. In 1997, vegetables and vegetable products were the primary contributors to total sugar, followed by cereals and cereals products, and dried legumes and legume products. In 2011, this list was topped with fresh fruits, cakes/pies/sweet rolls or other pastry, and vegetables and vegetable products. In 1997, dried legumes and legume products, vegetables and vegetable products, and sugars/candies were the most important dietary sources of added sugar. In 2011, cakes/pies/sweet rolls or other pastry, carbonated drinks, and ice-creams were the highest contributors to added sugar.

## 4. Discussion

As one of the most vulnerable groups, the excessive sugar intake observed among children and adolescents has aroused a wide public health concern. However, to our knowledge, only Afeiche, M.C. et al. assessed the sugar intakes of this population in 2011 [22]. Our study firstly reported the time trends in the total and added sugar intakes in Chinese 3-to-17-year-old children and adolescents covering 15 years.

In this study, despite the mean intakes of added sugar remaining below the DGA’s cut-off value until 2011, we observed a notable rise in intakes of total sugar, added sugar, total sugar (%E), and added sugar (%E), from 11.2 ± 0.3 g/d, 1.0 ± 0.1 g/d, 2.6 ± 0.1%, and 0.2 ± 0.0% in 1997 to 28.1 ± 0.5 g/d, 7.2 ± 0.3 g/d, 7.8 ± 0.1%, and 1.9 ± 0.1% in 2011, particularly since 2006. Our findings from the 2011 survey are supported by the estimates from Afeiche, M.C. et al. [22] in the same year (26 ± 0.6 g/d, 9 ± 0.3 g/d, 8 ± 0.1%, and 3 ± 0.1%). To explore the reasons for the upward trends in the total and added sugar intakes, the dietary sources of total and added sugars in 1997 and 2011 were analyzed. We found that the main dietary sources of total sugar were from naturally-occurring sources in 1997. Specifically, vegetables and vegetable products, cereals and cereals products, and dried legumes and legume products were the primary contributors to total sugar. In contrast, in 2011, cakes/pies/sweet rolls or other pastry was just below fresh fruits and had become the secondary contributor to total sugar. Regarding added sugar, dried legumes and legume products and vegetables and vegetable products were the most important dietary sources in 1997, while cakes/pies/sweet rolls or other pastry, carbonated drinks, and ice-creams were the highest contributors in 2011. These findings are consistent with Afeiche, M.C. et al. [22], who found that bakery products were common sources of total and added sugars among 4-to-13-year-old Chinese children in 2011. Based on our findings and the evidence of the upward trends in high-sugar foods such as sugar-sweetened beverages in the last few decades in China [23,24], we assumed the increased consumption of bakery products, carbonated drinks, and ice creams might be one of the main reasons for the increased sugar intake.

To facilitate the understanding of the total and added sugar intakes and time trends in children and adolescents with different demographics and characteristics, we also reported them by age groups, gender, area, and BMI categories. The data presented in this paper showed that adolescents aged 13–17 years had the highest total sugar intake, and children aged 3–6 years had the highest added sugar intake, except for 2011. Furthermore, children and adolescents living in urban areas and who were overweight had much higher total and added sugar intakes than those residing in rural areas and of non-overweight/obesity. It is important to note that the intakes of total sugar, added sugar, and added sugar (%E) were lower in obese children and adolescents than those in other BMI categories between 1997 and 2009, while the opposite was true in 2011, suggesting that the increased sugar intake might have gradually become an important contributor to obesity. Another potential explanation may be the low number of obese children or adolescents (*n* = 381), making the data unrepresentative. Additionally, our findings demonstrated the same upward trends in the total and added sugars intake by gender, area, and BMI categories in different age groups in the age-combined analysis. These results, on the one hand, indicate a negative shift in dietary quality among Chinese children and adolescents along with economic development, urban expansion and modernization, and improved living conditions, and on the other hand, confirm the necessity to implement strategies to reverse the rise of sugar intake among all young generations, particularly among those aged 3–6 years and 13–17 years, overweight and obese, as well as urban residents.

Worldwide, excessive sugar intake was found in children and adolescents around 2011 in many developed and developing countries [9,10,22,25]. However, both the United States and Germany have reported a stable decline in added sugar consumption after peaking over the past decade [9,10]. Several factors might be the main driving forces behind the reversal in the trends in sugar intakes which can be available for reference. These factors include the increasing awareness of high sugar intake levels [10], the growing concern regarding the rising obesity prevalence [26,27], the release of dietary guidelines providing a specific limit for sugars intake [1,28], the popularity of low-carbohydrate diets [9], and the lower added sugar and free sugar content in reformulated processed foods [10].

Actually, correct cognition and a healthy attitude toward sugars and related diseases are being promoted by the health education institutes in China [29]. The Dietary Guidelines for Chinese Residents (2016) [30] and the latest version (2022) [31] have proposed a specific limit on sugar intakes that control the daily added sugar intake to no more than 50 g, preferably less than 25 g. Importantly, improving dietary quality was incorporated into the action targets in the recent Healthy China 2030 national action plan [32]. With the support of such health education activities and national guidelines and policy, the growing trends in sugar intake in children and adolescents are expected to be curtailed as part of broader efforts to improve the health of the Chinese population. To make the healthy choices the easy choices in China, public health initiatives targeted at the population-level instead of at the individual-level will still be needed.

Besides providing data on the continued upward trends of total and added sugar intakes and changes in the diversity of their dietary sources among Chinese children and adolescents for the first time, the strengths of this study include its open-cohort design, allowing time–trend analyses in a large, nationally representative sample with a big sample size, and dietary intakes measured with three consecutive 24-h recalls and validated by an in-house weighing approach.

Our study was also subject to some limitations. First, since data on the total and added sugar contents in China Food Composition are still lacking, we used the SR28 and FPED 2015–2016, and combined the labeled ingredients and nutrient contents to estimate the total and added sugar intakes of Chinese children and adolescents, which may not be fully applied to Chinese foods due to the heterogeneity in food types between the two countries. However, the availability of the dietary data obtained by using the same or similar methods spanning 15 years made valid comparisons and the testing of trends possible. Second, because CHNS has not published dietary data after 2011 yet, the time trends of sugar intakes in recent years were not available in our study.

## 5. Conclusions

In conclusion, this study revealed continued growth trends in total and added sugar intakes among children and adolescents across all age groups in China over 15 years from 1997 to 2011. Adolescents aged 13–17 years had the highest total sugar intake, whereas children aged 3–6 years had the highest total sugar (%E), added sugar intake, and added sugar (%E). In terms of rural–urban differences, all of these sugar intake variables were much higher among children and adolescents living in urban areas than those residing in rural areas. In addition, children and adolescents who were overweight had higher total sugar, added sugar, total sugar (%E), and added sugar (%E) intakes than those of non-overweight/obesity. Furthermore, the dietary sources of the total and added sugars have become more diverse over the study period. These findings suggest that strategies regarding the macro-level treatment of the increasing dietary sugar intake in Chinese children and adolescents, especially in those aged 3–6 years, aged 13–17 years, overweight, obese, and urban residents, are urgently needed.

## Figures and Tables

**Table 1:** Basic demographics and characteristics of Chinese children and adolescents by years, CHNS ^1^ 1997–2011.

	1997 (*n* = 3078)	2000 (*n* = 2938)	2004 (*n* = 1950)	2006 (*n* = 1624)	2009 (*n* = 1544)	2011 (*n* = 2078)	Total (*n* = 13,212)
Characteristics (mean ± SE ^2^)							
Age (year)	10.4 ± 0.1	10.9 ± 0.1	10.6 ± 0.1	10.2 ± 0.1	9.6 ± 0.1	9.3 ± 0.1	10.2 ± 0.0
BMI ^3^ (kg/m^2^)	17.0 ± 0.1	17.3 ± 0.1	17.6 ± 0.1	17.3 ± 0.1	17.2 ± 0.1	17.9 ± 0.1	17.4 ± 0.0
Total energy intake (kcal/d)	1844.3 ± 11.0	1831.9 ± 11.6	1747.7 ± 15.0	1668.5 ± 15.7	1601.4 ± 15.1	1471.4 ± 12.6	1719.3 ± 5.5
Total fat intake (g/d)	51.0 ± 0.6	57.7 ± 0.7	54.6 ± 0.8	54.3 ± 0.8	56.8 ± 1.0	54.7 ± 0.7	54.7 ± 0.3
Total fat (%E ^4^)	24.8 ± 0.2	27.5 ± 0.2	27.5 ± 0.3	29.0 ± 0.3	31.0 ± 0.3	33.4 ± 0.3	28.4 ± 0.1
Total protein intake (g/d)	55.6 ± 0.6	55.4 ± 0.4	53.5 ± 0.6	50.8 ± 0.5	50.1 ± 0.5	50.2 ± 0.5	53.1 ± 0.2
Total protein (%E)	12.1 ± 0.0	12.2 ± 0.0	12.4 ± 0.1	12.3 ± 0.1	12.7 ± 0.1	14.0 ± 0.1	12.5 ± 0.0
Total carbohydrate intake (g/d)	289.8 ± 2.0	273.4 ± 1.9	256.3 ± 2.3	241.2 ± 2.6	221.5 ± 2.2	190.2 ± 1.9	251.6 ± 0.9
Total carbohydrate (%E)	63.2 ± 0.2	60.3 ± 0.2	60.1 ± 0.3	58.6 ± 0.3	56.3 ± 0.3	52.5 ± 0.3	59.1 ± 0.1
Total sugar intake (g/d)	11.2 ± 0.3	12.0 ± 0.2	15.9 ± 0.4	18.5 ± 0.5	22.2 ± 0.5	28.1 ± 0.5	16.9 ± 0.2
Total sugar (%E)	2.6 ± 0.1	2.8 ± 0.1	3.8 ± 0.1	4.7 ± 0.1	5.7 ± 0.1	7.8 ± 0.1	4.2 ± 0.0
Added sugar intake (g/d)	1.0 ± 0.1	1.0 ± 0.1	1.4 ± 0.1	2.1 ± 0.2	5.1 ± 0.3	7.2 ± 0.3	2.7 ± 0.1
Added sugar (%E)	0.2 ± 0.0	0.3 ± 0.0	0.3 ± 0.0	0.5 ± 0.0	1.3 ± 0.1	1.9 ± 0.1	0.7 ± 0.0
Annual household income (yuan)	14,051.4 ± 229.7	15,727.7 ± 310.5	19,888.5 ± 421.5	23,930.9 ± 822.5	38,682.6 ± 1110.3	52,384.7 ± 1262.5	25,395.3 ± 304.0
Characteristics (*n* (%))							
Gender							
Boys	1643 (53.4)	1563 (53.2)	1040 (53.3)	870 (53.6)	856 (55.4)	1080 (52.0)	7052 (53.4)
Girls	1435 (46.6)	1375 (46.8)	910 (46.7)	754 (46.4)	688 (44.6)	998 (48.0)	6160 (46.6)
Area of residence							
Urban	883 (28.8)	746 (27.8)	542 (28.1)	471 (29.4)	413 (26.9)	745 (36.2)	3800 (29.5)
Rural	2182 (71.2)	1940 (72.2)	1387 (71.9)	1131 (70.6)	1122 (73.1)	1313 (63.8)	9075 (70.5)
BMI categories							
Non-overweight/obesity	2527 (92.8)	2370 (92.0)	1548 (89.0)	1248 (88.3)	1191 (86.4)	1605 (81.2)	10,489 (88.8)
Overweight	154 (5.7)	162 (6.3)	145 (8.3)	118 (8.4)	135 (9.8)	223 (11.3)	937 (7.9)
Obesity	43 (1.6)	44 (1.7)	46 (2.6)	47 (3.3)	52 (3.8)	149 (7.5)	381 (3.2)
Medical insurance							
Yes	515 (17.1)	466 (16.6)	465 (24.3)	659 (41.1)	1323 (86.2)	1901 (92.4)	5329 (41.2)
No	2489 (82.9)	2334 (83.4)	1447 (75.7)	943 (58.9)	212 (13.8)	157 (7.6)	7582 (58.7)

Values are means ± standard errors (SEs). ^1^ CHNS: China Health and Nutrition Survey; ^2^ SE: standard error; ^3^ BMI: body mass index; ^4^ %E: % of energy. All results of linear trends by using general linear regression were *p* < 0.001.

**Table 2:** Comparisons of basic demographics and characteristics of Chinese children and adolescents with different total sugar intake, CHNS ^1^ 1997–2011.

	3–6 Years				7–12 Years				13–17 Years			
	Boys (*n* = 1553)		Girls (*n* = 1316)		Boys (*n* = 3164)		Girls (*n* = 2793)		Boys (*n* = 2335)		Girls (*n* = 2051)	
	Q1	Q2	Q1	Q2	Q1	Q2	Q1	Q2	Q1	Q2	Q1	Q2
Characteristics (mean ± SE ^2^)												
Age (year)	4.5 ± 0.0	4.6 ± 0.0	4.6 ± 0.0	4.6 ± 0.0	9.6 ± 0.0	9.7 ± 0.0 ^c^	9.6 ± 0.0	9.7 ± 0.0 ^c^	14.8 ± 0.0	14.8 ± 0.0	14.7 ± 0.0	14.8 ± 0.0
BMI ^3^ (kg/m^2^)	16.0 ± 0.1	16.4 ± 0.2 ^c^	15.6 ± 0.1	15.8 ± 0.1	16.5 ± 0.1	17.1 ± 0.1 ^a^	16.2 ± 0.1	16.7 ± 0.1 ^a^	18.9 ± 0.1	19.6 ± 0.1 ^a^	19.0 ± 0.1	19.5 ± 0.1 ^a^
Total energy intake (kcal/d)	1162.7 ± 14.6	1320.0 ± 16.7 ^a^	1134.0 ± 15.9	1224.6 ± 15.5 ^a^	1718.7 ± 13.7	1814.1 ± 13.9 ^a^	1599.3 ± 13.8	1670.6 ± 14.7 ^b^	2151.3 ± 17.8	2297.7 ± 19.8 ^a^	1866.3 ± 16.4	1878.9 ± 17.4
Total fat intake (g/d)	36.3 ± 0.8	46.5 ± 0.9 ^a^	34.9 ± 1.4	44.8 ± 0.9 ^a^	51.4 ± 0.8	60.6 ± 0.8 ^a^	48.2 ± 0.8	57.0 ± 0.9 ^a^	63.2 ± 1.1	73.5 ± 1.4 ^a^	56.1 ± 1.0	63.0 ± 1.2 ^a^
Total fat (%E ^4^)	28.4 ± 0.4	31.4 ± 0.4 ^a^	27.5 ± 0.5	32.5 ± 0.4 ^a^	26.5 ± 0.3	29.6 ± 0.3 ^a^	27.0 ± 0.3	30.2 ± 0.3 ^a^	25.8 ± 0.3	28.0 ± 0.3 ^a^	26.8 ± 0.4	29.4 ± 0.4 ^a^
Total protein intake (g/d)	34.0 ± 0.5	43.4 ± 0.6 ^a^	32.8 ± 0.6	39.8 ± 0.6 ^a^	51.0 ± 0.5	59.3 ± 0.9 ^a^	47.1 ± 0.5	53.3 ± 0.5 ^a^	64.4 ± 0.7	73.0 ± 0.8 ^a^	55.8 ± 0.7	60.5 ± 0.6 ^a^
Total protein (%E)	12.1 ± 0.1	13.4 ± 0.1 ^a^	12.1 ± 0.1	13.3 ± 0.1 ^a^	12.0 ± 0.1	13.0 ± 0.1 ^a^	11.9 ± 0.1	13.0 ± 0.1 ^a^	12.0 ± 0.1	12.8 ± 0.1 ^a^	12.0 ± 0.1	13.0 ± 0.1 ^a^
Total carbohydrate intake (g/d)	167.4 ± 2.4	178.6 ± 2.6 ^a^	163.7 ± 2.7	163.1 ± 2.4 ^a^	260.6 ± 2.3	260.2 ± 2.5	242.6 ± 2.4	235.9 ± 2.4 ^b^	332.4 ± 3.1	338.7 ± 3.5	283.8 ± 2.9	267.3 ± 2.8 ^a^
Total carbohydrate (%E)	59.5 ± 0.4	55.2 ± 0.4 ^a^	60.4 ± 0.5	54.2 ± 0.5 ^a^	61.5 ± 0.3	57.4 ± 0.3 ^a^	61.1 ± 0.3	56.8 ± 0.3 ^a^	62.1 ± 0.3	59.1 ± 0.3 ^a^	61.2 ± 0.4	57.5 ± 0.4 ^a^
Total sugar intake (g/d)	4.2 ± 0.1	28.2 ± 0.8 ^a^	4.1 ± 0.1	26.8 ± 0.7 ^a^	5.3 ± 0.1	28.5 ± 0.6 ^a^	5.2 ± 0.1	26.7 ± 0.6 ^a^	6.5 ± 0.1	31.1 ± 0.6 ^a^	5.9 ± 0.1	29.3 ± 0.6 ^a^
Total sugar (%E)	1.6 ± 0.0	8.9 ± 0.2 ^a^	1.6 ± 0.0	9.1 ± 0.2 ^a^	1.3 ± 0.0	6.7 ± 0.1 ^a^	1.4 ± 0.0	6.8 ± 0.1 ^a^	1.3 ± 0.0	5.7 ± 0.1 ^a^	1.4 ± 0.0	6.7 ± 0.2 ^a^
Added sugar intake (g/d)	0.2 ± 0.0	6.9 ± 0.5 ^a^	0.2 ± 0.0	6.3 ± 0.5 ^a^	0.2 ± 0.0	5.1 ± 0.3 ^a^	0.2 ± 0.0	4.5 ± 0.3 ^a^	0.2 ± 0.0	4.7 ± 0.3 ^a^	0.2 ± 0.0	4.4 ± 0.4 ^a^
Added sugar (%E)	0.1 ± 0.0	2.2 ± 0.1 ^a^	0.1 ± 0.0	2.1 ± 0.1 ^a^	0.0 ± 0.0	1.2 ± 0.1 ^a^	0.1 ± 0.0	1.1 ± 0.1 ^a^	0.0 ± 0.0	0.9 ± 0.1 ^a^	0.1 ± 0.0	1.0 ± 0.1 ^a^
Annual household income (yuan)	21,096.9 ± 1041.1	38,736.2 ± 1797.0 ^a^	20,570.2 ± 860.5	40,766.4 ± 1921.7 ^a^	19,415.4 ± 775.4	30,091.0 ± 980.8 ^a^	18,016.9 ± 544.7	28,481.1 ± 919.1 ^a^	18,257.0 ± 615.2	29,277.5 ± 1276.7 ^a^	17,960.6 ± 525.3	30,632.4 ± 1292.8 ^a^
Characteristics (*n* (%))												
Area of residence												
Urban	116 (15.3)	250 (32.8) ^a^	133 (20.8)	269 (41.2) ^a^	316 (20.3)	493 (31.7) ^a^	288 (21.2)	526 (32.8) ^a^	309 (27.7)	417 (36.9) ^a^	283 (29.0)	400 (40.1) ^a^
Rural	642 (84.7)	512 (67.2)	507 (79.2)	384 (58.8)	1243 (79.7)	1061 (68.3)	1068 (78.8)	848 (67.2)	805 (72.3)	714 (63.1)	694 (71.0)	597 (59.9)
BMI categories												
Non-overweight/obesity	586 (87.5)	569 (83.6)	484 (86.1)	470 (81.7)	1303 (90.0)	1202 (83.3) ^a^	1154 (91.4)	1110 (83.6) ^a^	948 (93.3)	920 (89.3) ^b^	876 (96.6)	867 (93.9) ^c^
Overweight	46 (6.9)	54 (7.9)	41 (7.3)	59 (10.3)	104 (7.2)	176 (12.2)	81 (6.4)	145 (7.9)	64 (6.3)	92 (8.9)	26 (2.9)	49 (5.3)
Obesity	38 (5.7)	58 (8.5)	37 (6.6)	46 (8.0)	41 (2.8)	65 (4.5)	27 (2.1)	35 (8.5)	4 (0.4)	18 (1.7)	5 (0.6)	7 (0.8)
Medical insurance												
Yes	281 (37.3)	443 (58.2) ^a^	217 (33.9)	399 (61.2) ^a^	474 (30.5)	837 (53.9) ^a^	443 (32.3)	678 (58.2) ^a^	296 (26.3)	515 (45.1) ^a^	292 (29.5)	454 (45.2) ^a^
No	473 (62.7)	318 (41.8)	423 (66.1)	253 (38.8)	1080 (69.5)	717 (46.1)	927 (67.7)	684 (41.8)	831 (73.7)	626 (54.9)	699 (70.5)	551 (54.8)

The participants were divided into Q1 and Q2 by the median value of total sugar intake of the corresponding characteristic subgroup. *p* values were calculated by Student’s *t*-tests or Mann–Whitney U test for continuous variables and chi-squared test for categorical variables. ^1^ CHNS: China Health and Nutrition Survey; ^2^ SE: standard error; ^3^ BMI: body mass index; ^4^ %E: % of energy. ^a,b,c^ indicate significant differences between Q1 and Q2 groups; ^a^ *p* < 0.001, ^b^ *p* < 0.01, ^c^ *p* < 0.05.

**Table 3:** Trends in total and added sugar intakes by gender, area, and BMI ^1^ categories among children and adolescents by age groups, CHNS ^2^ 1997–2011.

		1997	2000	2004	2006	2009	2011
3–6 years							
		*n* = 525	*n* = 482	*n* = 403	*n* = 377	*n* = 445	*n* = 637
Gender							
Boys	Total sugar intake (g/d)	9.5 ± 0.8	10.1 ± 0.8	13.1 ± 1.4	16.2 ± 1.3	19.8 ± 1.3	25.8 ± 1.4
	Total sugar (%E ^3^)	2.9 ± 0.3	3.1 ± 0.2	4.7 ± 0.4	5.6 ± 0.4	6.6 ± 0.4	8.3 ± 0.3
	Added sugar intake (g/d)	1.2 ± 0.3	2.1 ± 0.6	1.9 ± 0.8	2.8 ± 0.6	5.2 ± 0.8	6.9 ± 0.6
	Added sugar (%E)	0.4 ± 0.1	0.6 ± 0.2	0.7 ± 0.3	0.9 ± 0.2	1.7 ± 0.2	2.2 ± 0.2
Girls	Total sugar intake (g/d)	9.6 ± 0.7	8.9 ± 0.7	11.1 ± 0.8	15.1 ± 1.4	21.1 ± 1.5	24.4 ± 1.1
	Total sugar (%E)	3.1 ± 0.2	3.1 ± 0.2	4.1 ± 0.3	5.6 ± 0.5	7.0 ± 0.4	9.1 ± 0.4
	Added sugar intake (g/d)	1.4 ± 0.3	0.7 ± 0.2	0.8 ± 0.2	2.5 ± 0.6	6.3 ± 1.1	6.8 ± 0.6
	Added sugar (%E)	0.5 ± 0.1	0.2 ± 0.1	0.3 ± 0.1	0.9 ± 0.2	1.9 ± 0.3	2.4 ± 0.2
Area							
Urban	Total sugar intake (g/d)	15.8 ± 1.5	12.4 ± 1.4	19.9 ± 2.5	22.7 ± 2.4	26.0 ± 2.0	34.5 ± 1.9
	Total sugar (%E)	4.6 ± 0.4	4.0 ± 0.4	6.8 ± 0.9	7.6 ± 0.8	8.3 ± 0.5	11.9 ± 0.5
	Added sugar intake (g/d)	3.0 ± 0.7	1.3 ± 0.4	4.0 ± 1.7	4.5 ± 1.2	8.9 ± 1.6	9.5 ± 0.9
	Added sugar (%E)	0.9 ± 0.2	0.5 ± 0.1	1.5 ± 0.6	1.4 ± 0.3	2.5 ± 0.4	3.2 ± 0.3
Rural	Total sugar intake (g/d)	7.3 ± 0.5	8.4 ± 0.6	9.7 ± 0.7	12.9 ± 0.9	18.4 ± 1.1	20.8 ± 1.0
	Total sugar (%E)	2.4 ± 0.2	2.8 ± 0.2	3.7 ± 0.2	4.8 ± 0.3	6.2 ± 0.3	7.2 ± 0.3
	Added sugar intake (g/d)	0.7 ± 0.1	1.2 ± 0.4	0.6 ± 0.2	1.9 ± 0.3	4.6 ± 0.7	5.7 ± 0.5
	Added sugar (%E)	0.2 ± 0.1	0.4 ± 0.1	0.2 ± 0.1	0.7 ± 0.1	1.5 ± 0.2	1.9 ± 0.1
BMI ^1^ categories							
Non-overweight/obesity	Total sugar intake (g/d)	10.0 ± 0.7	9.1 ± 0.6	12.1 ± 0.9	15.4 ± 1.0	20.4 ± 1.2	24.2 ± 0.9
	Total sugar (%E)	3.1 ± 0.2	2.9 ± 0.2	4.2 ± 0.3	5.5 ± 0.4	6.8 ± 0.3	8.7 ± 0.3
	Added sugar intake (g/d)	1.4 ± 0.2	1.2 ± 0.3	1.3 ± 0.2	2.9 ± 0.5	5.9 ± 0.8	6.5 ± 0.5
	Added sugar (%E)	0.4 ± 0.1	0.4 ± 0.1	0.4 ± 0.1	1.0 ± 0.2	1.8 ± 0.2	2.2 ± 0.2
Overweight	Total sugar intake (g/d)	9.5 ± 1.8	8.1 ± 1.8	13.3 ± 2.2	14.0 ± 3.5	26.0 ± 3.9	24.8 ± 2.3
	Total sugar (%E)	3.0 ± 0.6	3.1 ± 0.8	4.8 ± 0.9	4.5 ± 1.0	8.5 ± 1.1	9.0 ± 0.8
	Added sugar intake (g/d)	1.2 ± 0.8	0.6 ± 0.6	0.6 ± 0.4	1.4 ± 0.9	5.8 ± 1.9	7.7 ± 1.4
	Added sugar (%E)	0.4 ± 0.2	0.2 ± 0.2	0.3 ± 0.2	0.5 ± 0.3	2.3 ± 0.9	2.6 ± 0.5
Obesity	Total sugar intake (g/d)	7.7 ± 2.0	12.0 ± 3.1	10.9 ± 1.6	15.3 ± 2.7	14.0 ± 2.1	32.7 ± 4.6
	Total sugar (%E)	2.1 ± 0.6	3.9 ± 1.1	4.1 ± 0.6	5.8 ± 1.1	4.6 ± 0.6	9.0 ± 0.9
	Added sugar intake (g/d)	0.3 ± 0.3	1.5 ± 1.0	0.5 ± 0.3	0.9 ± 0.5	4.0 ± 1.3	9.5 ± 1.5
	Added sugar (%E)	0.1 ± 0.1	0.3 ± 0.2	0.2 ± 0.1	0.3 ± 0.2	1.4 ± 0.5	2.7 ± 0.4
7–12 years							
		*n* = 1567	*n* = 1347	*n* = 779	*n* = 710	*n* = 666	*n* = 888
Gender							
Boys	Total sugar intake (g/d)	11.4 ± 0.6	11.7 ± 0.4	14.7 ± 0.8	18.4 ± 1.0	23.7 ± 1.2	30.6 ± 1.2
	Total sugar (%E)	2.6 ± 0.1	2.7 ± 0.1	3.4 ± 0.2	4.5 ± 0.2	5.4 ± 0.2	7.7 ± 0.3
	Added sugar intake (g/d)	0.9 ± 0.1	0.8 ± 0.1	0.9 ± 0.2	2.0 ± 0.4	5.5 ± 0.6	8.2 ± 0.9
	Added sugar (%E)	0.2 ± 0.0	0.2 ± 0.0	0.2 ± 0.0	0.5 ± 0.1	1.2 ± 0.1	1.9 ± 0.2
Girls	Total sugar intake (g/d)	11.5 ± 0.7	11.8 ± 0.5	15.6 ± 0.8	16.8 ± 0.9	21.1 ± 1.3	25.5 ± 1.1
	Total sugar (%E)	2.8 ± 0.2	2.8 ± 0.1	3.9 ± 0.2	4.4 ± 0.2	5.4 ± 0.3	7.4 ± 0.3
	Added sugar intake (g/d)	1.0 ± 0.2	1.1 ± 0.2	1.4 ± 0.3	1.7 ± 0.3	5.0 ± 0.8	6.0 ± 0.5
	Added sugar (%E)	0.2 ± 0.0	0.2 ± 0.1	0.3 ± 0.1	0.4 ± 0.1	1.2 ± 0.2	1.7 ± 0.1
Area							
Urban	Total sugar intake (g/d)	17.8 ± 1.5	15.0 ± 0.9	19.3 ± 1.3	23.8 ± 1.7	31.4 ± 2.0	34.3 ± 1.6
	Total sugar (%E)	3.9 ± 0.3	3.4 ± 0.2	4.4 ± 0.3	5.7 ± 0.4	7.4 ± 0.4	9.2 ± 0.3
	Added sugar intake (g/d)	1.9 ± 0.3	1.5 ± 0.3	2.4 ± 0.4	3.5 ± 0.7	9.1 ± 1.4	9.5 ± 1.2
	Added sugar (%E)	0.4 ± 0.1	0.4 ± 0.1	0.5 ± 0.1	0.8 ± 0.1	2.0 ± 0.3	2.3 ± 0.2
Rural	Total sugar intake (g/d)	8.9 ± 0.2	10.6 ± 0.3	13.8 ± 0.6	15.4 ± 0.7	19.7 ± 0.9	24.8 ± 0.9
	Total sugar (%E)	2.2 ± 0.1	2.5 ± 0.1	3.4 ± 0.1	4.0 ± 0.2	4.8 ± 0.2	6.6 ± 0.2
	Added sugar intake (g/d)	0.6 ± 0.1	0.8 ± 0.2	0.7 ± 0.2	1.3 ± 0.2	4.1 ± 0.5	5.8 ± 0.5
	Added sugar (%E)	0.1 ± 0.0	0.2 ± 0.0	0.2 ± 0.0	0.3 ± 0.1	0.9 ± 0.1	1.5 ± 0.1
BMI categories							
Non-overweight/obesity	Total sugar intake (g/d)	11.4 ± 0.5	11.6 ± 0.4	14.4 ± 0.6	16.8 ± 0.7	22.3 ± 1.0	27.1 ± 0.9
	Total sugar (%E)	2.6 ± 0.1	2.7 ± 0.1	3.5 ± 0.1	4.3 ± 0.2	5.4 ± 0.2	7.5 ± 0.2
	Added sugar intake (g/d)	0.9 ± 0.1	1.0 ± 0.2	1.0 ± 0.2	1.8 ± 0.3	5.4 ± 0.6	6.4 ± 0.5
	Added sugar (%E)	0.2 ± 0.0	0.2 ± 0.0	0.2 ± 0.0	0.4 ± 0.1	1.2 ± 0.1	1.7 ± 0.1
Overweight	Total sugar intake (g/d)	15.8 ± 2.8	14.0 ± 1.2	17.4 ± 2.3	24.6 ± 3.0	30.2 ± 2.8	29.6 ± 2.0
	Total sugar (%E)	3.4 ± 0.5	3.0 ± 0.2	4.1 ± 0.5	5.6 ± 0.6	6.9 ± 0.6	7.5 ± 0.4
	Added sugar intake (g/d)	1.7 ± 0.6	1.8 ± 0.6	2.3 ± 0.9	3.6 ± 1.2	6.8 ± 1.6	8.4 ± 1.2
	Added sugar (%E)	0.4 ± 0.1	0.3 ± 0.1	0.5 ± 0.2	0.8 ± 0.2	1.5 ± 0.3	2.0 ± 0.3
Obesity	Total sugar intake (g/d)	9.7 ± 1.4	12.1 ± 3.2	21.0 ± 5.6	19.4 ± 3.3	20.6 ± 3.9	37.6 ± 5.4
	Total sugar (%E)	3.0 ± 0.6	2.6 ± 0.5	4.6 ± 0.9	5.6 ± 0.9	5.8 ± 1.3	8.8 ± 0.9
	Added sugar intake (g/d)	0.3 ± 0.2	0.2 ± 0.1	2.0 ± 1.0	1.2 ± 0.5	3.2 ± 1.3	13.6 ± 4.9
	Added sugar (%E)	0.0 ± 0.0	0.0 ± 0.0	0.3 ± 0.2	0.4 ± 0.2	1.0 ± 0.4	2.8 ± 0.8
13–17 years							
		*n* = 986	*n* = 1109	*n* = 768	*n* = 537	*n* = 433	*n* = 553
Gender							
Boys	Total sugar intake (g/d)	11.4 ± 0.5	14.0 ± 0.5	20.2 ± 1.1	22.4 ± 1.3	24.2 ± 1.4	32.4 ± 1.5
	Total sugar (%E)	2.0 ± 0.1	2.6 ± 0.1	3.6 ± 0.2	4.0 ± 0.2	4.6 ± 0.3	6.5 ± 0.3
	Added sugar intake (g/d)	0.9 ± 0.2	1.0 ± 0.2	1.7 ± 0.3	2.6 ± 0.5	4.0 ± 0.6	8.1 ± 0.8
	Added sugar (%E)	0.1 ± 0.0	0.2 ± 0.0	0.3 ± 0.0	0.5 ± 0.1	0.8 ± 0.1	1.5 ± 0.1
Girls	Total sugar intake (g/d)	12.2 ± 0.7	12.7 ± 0.6	16.8 ± 0.8	20.8 ± 1.1	22.8 ± 1.5	30.5 ± 1.5
	Total sugar (%E)	2.6 ± 0.2	2.8 ± 0.1	3.7 ± 0.2	4.7 ± 0.3	5.4 ± 0.4	7.9 ± 0.3
	Added sugar intake (g/d)	1.1 ± 0.3	1.0 ± 0.2	1.4 ± 0.3	1.6 ± 0.4	4.4 ± 1.0	7.1 ± 0.8
	Added sugar (%E)	0.2 ± 0.1	0.2 ± 0.1	0.3 ± 0.1	0.3 ± 0.1	1.0 ± 0.2	1.7 ± 0.2
Area							
Urban	Total sugar intake (g/d)	15.8 ± 1.1	14.6 ± 0.8	22.4 ± 1.6	26.6 ± 1.9	29.6 ± 2.1	35.5 ± 1.7
	Total sugar (%E)	3.1 ± 0.3	2.9 ± 0.2	4.2 ± 0.2	5.2 ± 0.3	6.3 ± 0.5	8.5 ± 0.4
	Added sugar intake (g/d)	2.1 ± 0.5	1.5 ± 0.3	2.5 ± 0.4	4.1 ± 0.8	7.0 ± 1.4	9.7 ± 0.9
	Added sugar (%E)	0.4 ± 0.1	0.3 ± 0.1	0.5 ± 0.1	0.7 ± 0.1	1.4 ± 0.3	2.2 ± 0.2
Rural	Total sugar intake (g/d)	10.0 ± 0.4	13.1 ± 0.5	16.9 ± 0.7	19.1 ± 0.9	20.9 ± 1.1	28.4 ± 1.4
	Total sugar (%E)	1.9 ± 0.1	2.6 ± 0.1	3.4 ± 0.1	3.9 ± 0.2	4.4 ± 0.2	6.2 ± 0.3
	Added sugar intake (g/d)	0.5 ± 0.1	0.8 ± 0.1	1.1 ± 0.2	1.1 ± 0.2	2.9 ± 0.4	6.0 ± 0.8
	Added sugar (%E)	0.1 ± 0.0	0.2 ± 0.0	0.2 ± 0.0	0.2 ± 0.0	0.6 ± 0.1	1.2 ± 0.1
BMI categories							
Non-overweight/obesity	Total sugar intake (g/d)	11.9 ± 0.5	13.2 ± 0.4	18.3 ± 0.8	21.5 ± 1.0	23.0 ± 1.1	31.6 ± 1.2
	Total sugar (%E)	2.3 ± 0.1	2.7 ± 0.1	3.6 ± 0.1	4.3 ± 0.2	4.9 ± 0.2	7.3 ± 0.2
	Added sugar intake (g/d)	1.1 ± 0.2	1.0 ± 0.1	1.7 ± 0.2	2.4 ± 0.4	4.0 ± 0.6	7.6 ± 0.7
	Added sugar (%E)	0.2 ± 0.0	0.2 ± 0.0	0.3 ± 0.0	0.4 ± 0.1	0.9 ± 0.1	1.6 ± 0.1
Overweight	Total sugar intake (g/d)	16.4 ± 2.5	19.4 ± 2.9	17.4 ± 2.3	25.3 ± 4.5	23.7 ± 3.3	32.4 ± 3.6
	Total sugar (%E)	3.1 ± 0.5	3.4 ± 0.4	3.4 ± 0.5	4.8 ± 0.8	4.7 ± 0.6	6.5 ± 0.6
	Added sugar intake (g/d)	0.9 ± 0.4	1.9 ± 1.1	1.4 ± 0.8	1.2 ± 0.5	4.5 ± 1.7	7.5 ± 1.5
	Added sugar (%E)	0.1 ± 0.1	0.3 ± 0.1	0.3 ± 0.2	0.2 ± 0.1	1.0 ± 0.4	1.4 ± 0.2
Obesity	Total sugar intake (g/d)	13.5 ± 1.8	11.0 ± 6.4	17.1 ± 4.0	6.3 ± 0.8	35.3 ± 7.5	37.9 ± 7.4
	Total sugar (%E)	3.2 ± 1.6	1.7 ± 0.8	3.1 ± 1.1	1.4 ± 0.1	6.7 ± 0.9	8.0 ± 1.3
	Added sugar intake (g/d)	0.8 ± 0.5	0.4 ± 0.3	0.0 ± 0.0	0.4 ± 0.4	7.4 ± 7.0	7.4 ± 2.6
	Added sugar (%E)	0.1 ± 0.1	0.1 ± 0.1	0.0 ± 0.0	0.1 ± 0.1	0.9 ± 0.8	1.4 ± 0.5

Values are means ± standard errors (SEs). ^1^ BMI: body mass index; ^2^ CHNS: China Health and Nutrition Survey; ^3^ %E: % of energy. All results of linear trends by using general linear regression were *p* < 0.001.

**Table 4:** Dietary sources of total and added sugars among Chinese children and adolescents, CHNS ^1^ 1997 and 2011.

**Dietary Sources of Total Sugar**					
**1997**			**2011**		
**Rank**	**Food/Beverage Groups**	**% of Total Intake**	**Rank**	**Food/Beverage Groups**	**% of Total Intake**
1	Vegetables and vegetable products	34.3	1	Fresh fruits	22.9
2	Cereals and cereals products	32.1	2	Cakes/pies/sweet rolls or other pastry	20.3
3	Dried legumes and legume products	23.7	3	Vegetables and vegetable products	15.2
4	Tubers/starches products	6.8	4	Liquid milk	12.0
5	Sugars/Candies	1.6	5	Cereals and cereals products	5.0
6	Others ^2^	1.3	6	Dried legumes and legume products	4.0
			7	Ice-creams	2.4
			8	Carbonated drinks	2.3
			9	Biscuits	2.2
			10	Quick bread and bread products	2.1
			11	Sugars/Candies	1.8
			12	Yogurt	1.6
			13	Infant foods	1.4
			14	Fruit Juices and drinks	1.3
			15	Tubers/starches products	1.1
			16	Nuts and seeds	1.1
			17	Others ^2^	3.4
**Dietary Sources of Added Sugar**					
1997			2011		
Rank	Food/beverage groups	% of total intake	Rank	Food/beverage groups	% of total intake
1	Dried legumes and legume products	68.1	1	Cakes/pies/sweet rolls or other pastry	56.8
2	Vegetables and vegetable products	13.9	2	Carbonated drinks	7.7
3	Sugars/Candies	11.2	3	Ice-creams	6.8
4	Biscuits	4.9	4	Biscuits	6.4
5	Quick bread and bread products	1.9	5	Sugars/Candies	6.1
			6	Quick bread and bread products	4.6
			7	Infant foods	4.4
			8	Fruit Juices and drinks	3.4
			9	Dried legumes and legume products	1.8
			10	Vegetable protein drinks	1.1
			11	Others ^2^	0.9

^1^ CHNS: China Health and Nutrition Survey. ^2^ Include food or beverage groups that contribute to less than 1% of total or added sugar intakes.

## Data Availability

Publicly available datasets were analyzed in this study. This data can be found here: [https://www.cpc.unc.edu/projects/china/data].

## Supplementary Materials

The following supporting information can be downloaded at: https://www.mdpi.com/article/10.3390/nu14163340/s1, Table S1: Percentage of the children and adolescents below and above or equal to 10% of total energy intake from added sugar by age, gender, area, and BMI categories, CHNS 1997–2011.
