# Supplementary material for: Total and Added Sugar Intakes Are Increasing among Children and Adolescents in China: Findings from CHNS 1997–2011
Source: Nutrients. 2022 Aug 15;14(16):3340. doi: 10.3390/nu14163340 (PMC9416199; doi:10.3390/nu14163340)
Supplement: Supplementary file 1 [file nutrients-14-03340-s001.zip › nutrients-1829804-supplementary.pdf]

Percentage of the children and adolescents below and above or equal to 10% of total energy intake from added sugar by age, gender, area, and BMI categories

The percentages of the children and adolescents below and above or equal to 10% of total energy intake from added sugar by age, gender, area, and BMI categories from 1997 to 2011 are shown in Table S1. The majority of Chinese children and adolescents were below the DGA's cut-off value for added sugar intake. However, the percentages above or equal to this cut-off value increased among all subgroups over the study period.

Table S1. Percentage of the children and adolescents below and above or equal to 10% of total energy intake from added sugar by age, gender, area, and BMI[1] categories, CHNS[1] 1997–2011

		1997 (n=3078)	2000 (n=2938)	2004 (n=1950)	2006 (n=1624)	2009 (n=1544)	2011 (n=2078)
Age groups	Added sugar (%E[1]) categories						
3-6 years	< 10%	513(99.4)	472(99.0)	379(99.2)	357(98.1)	415(96.1)	584(96.2)
	≥ 10%	3(0.6)	5(1.0)	3(0.8)	7(1.9)	17(3.9)	23(3.8)
7-12 years	< 10%	1552(99.8)	1341(99.7)	776(99.9)	706(99.6)	653(98.0)	856.0(96.9)
	≥ 10%	3(0.2)	4(0.3)	1(0.1)	3(0.4)	13(2.0)	27(3.1)
13-17 years	< 10%	984(99.9)	1102(99.7)	767(99.9)	536(99.8)	426(98.8)	535(97.6)
	≥ 10%	1(0.1)	3(0.3)	1(0.1)	1(0.2)	5(1.2)	13(2.4)
Gender	Added sugar (%E) categories						
Boys	< 10%	1630(99.9)	1548(99.5)	1024(99.7)	858(99.3)	829(97.9)	1031(97.1)
	≥ 10%	2(0.1)	7(0.5)	3(0.3)	6(0.7)	18(2.1)	31(2.9)
Girls	< 10%	1419(99.6)	1367(99.6)	898(99.8)	741(99.3)	665(97.5)	944(96.7)
	≥ 10%	5(0.4)	5(0.4)	2(0.2)	5(0.7)	17(2.5)	32(3.3)
Area	Added sugar (%E) categories						
Urban	< 10%	874(99.4)	740(99.5)	534(99.6)	463(98.5)	396(96.6)	699(95.4)
	≥ 10%	5(0.6)	4(0.5)	2(0.4)	7(1.5)	14(3.4)	34(4.6)
Rural	< 10%	2162(99.9)	1926(99.7)	1367(99.8)	1115(99.6)	1089(98.1)	1258(97.8)
	≥ 10%	2(0.1)	6(0.3)	3(0.2)	4(0.4)	21(1.9)	28(2.2)
BMI categories	Added sugar (%E) categories						
Non-overweight/obesity	< 10%	2504(99.8)	2352(99.6)	1528(99.8)	1233(99.4)	1152(97.6)	1529(97.1)
	≥ 10%	6(0.2)	9(0.4)	3(0.2)	8(0.6)	28(2.4)	46(2.9)
Overweight	< 10%	154(100.0)	162(100.0)	143(99.3)	117(99.2)	131(97.0)	212(96.4)
	≥ 10%	0(0.0)	0(0.0)	1(0.7)	1(0.8)	4(3.0)	8(3.6)
Obesity	< 10%	43(100.0)	44(100.0)	46(100.0)	46(100.0)	52(100.0)	139(95.2)
	≥ 10%	0(0.0)	0(0.0)	0(0.0)	0(0.0)	0(0.0)	7(4.8)

[1] BMI: body mass index; CHNS: China Health and Nutrition Survey; % E: % of energy.
